# An Effective Microwave-Induced Iodine-Catalyzed Method for the Synthesis of Quinoxalines via Condensation of 1,2-Diamines with 1,2-Dicarbonyl Compounds

**DOI:** 10.3390/molecules15064207

**Published:** 2010-06-09

**Authors:** Debasish Bandyopadhyay, Sanghamitra Mukherjee, Robert R. Rodriguez, Bimal K. Banik

**Affiliations:** Department of Chemistry, The University of Texas-Pan American, 1201 West University Drive, Edinburg, TX 78541, USA

**Keywords:** iodine, microwave, quinoxalines, amines, dicarbonyl compounds

## Abstract

A microwave-induced iodine-catalyzed simple, rapid and convenient synthesis of different types of quinoxalines via condensation of 1,2-diamines with 1,2-dicarbonyl compounds has been accomplished with an excellent yield.

## 1. Introduction

A quinoxaline moiety serves as the nucleus for the synthesis of several biologically active compounds including antitumor [1], antimycobacterial [2] and antidepressant [3] drugs. Some antibiotics, such as levomycin, actinoleutin and echinomycin also contain a quinoxaline scaffold and these are known to inhibit the growth of Gram positive bacteria [4] and are active against various transplantable tumors [5]. As a part of our ongoing research leading to the synthesis of novel anticancer drugs [6,7,8,9,10,11] we became interested in the development of a new, efficient and practical method to synthesize quinoxalines. 

**Scheme 1:**
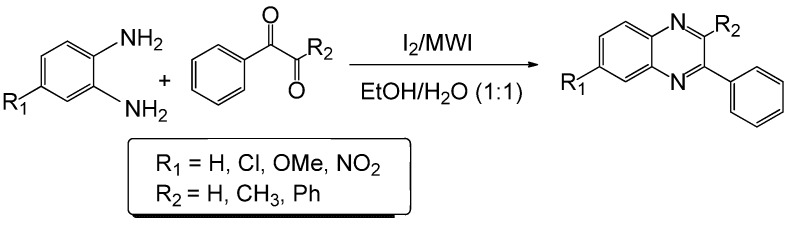
Microwave-induced iodine-catalyzed synthesis of quinoxalines.

Although diverse methods for the synthesis of substituted quinoxalines are described in the literature [12,13], the most common method for their preparation is a condensation reaction between a 1,2-diamine and 1,2 dicarbonyl compound. Clearly, all while these methods have extended the scope for the synthesis of this type of heterocycles, they have limitations in some of the following areas: low yield, long reaction time, difficult product isolation procedure and use of toxic metal catalysts as well as hazardous solvents. In this paper, we describe an automated microwave-assisted extremely rapid iodine-catalyzed method for the synthesis of substituted quinoxalines in high yields in aqueous ethanol *via* the diamine-dicarbonyl compound condensation route (Scheme 1).

## 2. Results and Discussion

Our group has reported the synthesis and biological evaluation of various β-lactams as novel anticancer agents [6,7,8,9,10,11]. The synthesis of β-lactams through imines requires a dicarbonyl compound and an amine. We have been conducting research on imines for the last 20 years. Our study suggests that condensation of 1,2-diamino compounds and 1,2 dicarbonyl compounds in the presence of a mild acidic reagent will lead to the synthesis of quinaxolines (Scheme 1). This hypothesis has been tested by reacting several diamines with various dicarbonyl compounds in the presence of iodine (catalytic amount, 5 mol %) in ethanol/water (1:1) using a microwave-induced method. The model reaction between *o*-phenylenediamine and phenylglyoxal monohydrate in the presence of iodine as catalyst using microwave irradiation has been performed in different solvent systems (Table 1). However, best results were obtained when EtOH/H_2_O (1:1) was used as solvent. 

**Table 1:** Reaction between *o*-phenylene diamine and phenylglyoxal monohydrate in the presence of iodine (5 mol%) in different solvents under microwave irradiation. 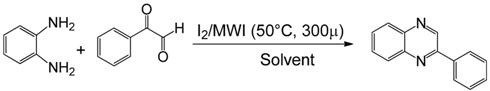

Entry	Solvent	Time (min.)	Yield (%)
1	Methanol	3	41
2	Ethanol	3	65
3	Tetrahydrofuran	5	48
4	Dichloromethane	3	27
5	THF/Water (1:1)	5	36
6	Water	5	38
7	Acetonitrile	3	72
8	Ethanol/Water (1:1)	30 sec.	99

Next the reaction conditions were tested with a variety of 1,2-diamines and 1,2-dicarbonyl compounds. In all cases the reaction was completed within 2–3 minutes at 50 °C in a CEM microwave and the corresponding quinoxalines were isolated in excellent yields (Table 2). 

**Table 2:** Synthesis of quinoxalines via condensation of 1,2-diamines with 1,2-dicarbonyl compounds with iodine as the catalyst in water/ethanol (1:1) under microwave induces reaction (Scheme 1).

Entry	Amine	Dicarbonyl Compounds	MWI (Power/temp/time)	Product	Yield (%)^a^
1	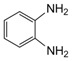	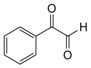	300 watts 50°C 30 sec	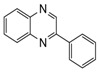	99
2	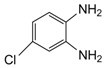	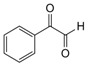	300 watts 50 °C 30 sec	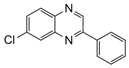	90
3	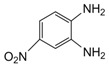	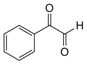	300 watts 50 °C 30 sec	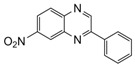	92
4	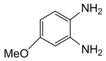	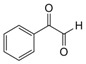	300 watts 50 °C 45 sec	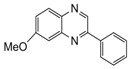	95
5	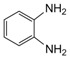	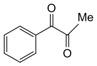	300 watts 50 °C 1 min	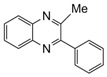	98
6	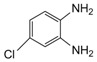	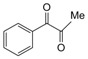	300 watts 50 °C 1 min	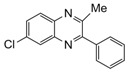	86
7	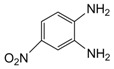	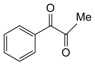	300 watts 50 °C 1.5 min	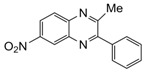	90
8	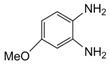	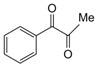	300 watts 50 °C 30 sec	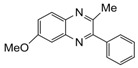	85
9	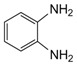	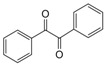	300 watts 50 °C 2.5 min	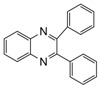	95
10	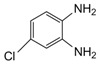	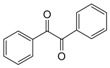	300 watts 50 °C 2 min	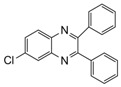	87
11	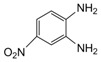	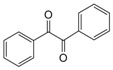	300 watts 50 °C 2 min 15 sec	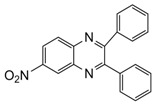	92
12	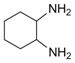	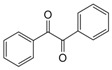	300 watts 50 °C 45 sec	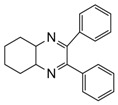	93
13	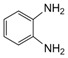	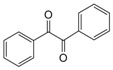	300 watts 50 °C 5 min without catalyst	No reaction	—

^a^ isolated yield.

The reaction proceeded equally well irrespective of the nature of substituents (chloro, nitro and methoxy groups) present in the amino component. The reaction between 1,2-diamines and 1,2 dicarbonyl compounds does not proceed at all without iodine (Entry 13).

## 3. Experimental Section

### 3.1. General

Melting points were determined in a Fisher Scientific electrochemical Mel-Temp* manual melting point apparatus (Model 1001) equipped with a 300°C thermometer. Elemental analyses (C, H, N) were conducted using the Perkin-Elmer 2400 series II elemental analyzer, their results were found to be in good agreement (± 0.2%) with the calculated values for C, H, N. FT-IR spectra were registered on a Bruker IFS 55 Equinox FTIR spectrophotometer as KBr discs. ^1^H-NMR (300 MHz) and ^13^C-NMR (75.4 MHz) spectra were obtained at room temperature with JEOL Eclipse-300 equipment using TMS as internal standard and CDCl_3_ as solvent. Analytical grade chemicals (Sigma-Aldrich incorporation) were used throughout the project. Deionized water was used for the preparation of all aqueous solutions.

### 3.2. General procedure for the synthesis of quinoxalines

The diamine (1 mmol) and the dicarbonyl compound (1 mmol) were dissolved in ethanol/water (1:1, 1 mL). A catalytic amount (5 mol%) of iodine was added and the mixture was irradiated using a CEM microwave (50 °C and power level 300 µ) as described in Table 2. The reaction was monitored by TLC. After the completion of the reaction, dichloromethane (10 mL) was added to the reaction mixture and it was then washed successively with 5% sodium thiosulphate solution (2 mL) and brine (2 mL). The organic layer was dried with anhydrous sodium sulfate and concentrated. Quinoxalines were found to be. No column chromatography was needed for the purification of the products and pure products (~95% by proton NMR) were isolated through crystallization (dichloromethane-hexane). Compounds obtained from entries 5, 6 and 9–11 (Table 2) were previously reported [13,14,15]. The remaining compounds (entries 1–4, 7, 8 and 12, Table 2) have been characterized by IR, NMR and mass spectral data. Our products gave satisfactory spectral and mp data matching with the reported values. We also carried out a reaction of benzil and *ortho*-phenylenediamine in absence of iodine (entry 13) following the published procedure [14]. However, no reaction took place at 50 °C without catalyst, even after 5 minutes of microwave irradiation; thus suggesting a lowering of the activation energy in the presence of the catalyst that also reduces the possibility of product loss due to partial charring.

## 4. Conclusions

In conclusion, the iodine-catalyzed microwave-induced quinoxaline synthesis as described herein is very simple, environmentally friendly and extremely rapid and affords high (almost quantitative) yields. Our method is versatile since aliphatic and aromatic amino compounds can be used in this reaction and these substrates all produce quinoxalines with remarkable success. 
